# Prediction of Diabetes Among Homeless Adults Using Artificial Intelligence: Suggested Recommendations

**DOI:** 10.3390/healthcare14060808

**Published:** 2026-03-22

**Authors:** Khadraa Mohamed Mousa, Farid Ali Mousa, Naglaa Mahmoud Abdelhamid, Mona Sayed Atress, Amal Yousef Abdelwahed, Olfat Yousef Gushgari, Fadiyah Alshwail, Rowaedh Ahmed Bawaked, Manal Mohamed Elsawy

**Affiliations:** 1 Community Health Nursing Department, Faculty of Nursing, Cairo University, Cairo 12613, Egypt; monaatress60@gmail.com (M.S.A.); manalelsawy@cu.edu.eg (M.M.E.); 2 Information Technology Department, Faculty of Computers and Artificial Intelligence, Beni-Suef University, Beni-Suef 62511, Egypt; faly@msa.edu.eg; 3 Gerontological Nursing Department, Faculty of Nursing, Cairo University, Cairo 12613, Egypt; naglaamahmoud_20@yahoo.com; 4 Public Health Department, College of Health Sciences, Saudi Electronic University, Riyadh 11673, Saudi Arabia; a.elnabasy@seu.edu.sa (A.Y.A.); o.gushgari@seu.edu.sa (O.Y.G.); f.alshwail@seu.edu.sa (F.A.); r.bawaked@seu.edu.sa (R.A.B.)

**Keywords:** diabetes prediction, homeless population, artificial intelligence, machine learning, healthcare recommendations

## Abstract

**Background:** Diabetes mellitus is a global health challenge, especially among homeless people. Early prediction of diabetes can reduce treatment costs and improve interventions. This study aimed to identify predictors of diabetes among homeless adults by utilizing artificial intelligence and providing recommendations for diabetes prevention. **Methods:** A case-control study of 150 homeless adults in Giza, Egypt (99 diabetes cases and 51 controls), analyzed 43 variables collected through interviews and physiological measures, with missing data imputed. Feature selection using recursive feature elimination and univariate and correlation analyses reduced the predictors to 13 variables. The class imbalance was addressed using synthetic minority over-sampling on the training set. Six models and a stacking ensemble with XGBoost as a meta-learner were evaluated using 5-fold cross-validation and performance metrics, including the accuracy, precision, recall, F1-score, and AUC-ROC. **Results:** The key predictors included BMI, systolic blood pressure, triceps skinfold thickness, waist circumference, lifestyle factors, comorbidities, diastolic blood pressure, age, medication adherence, educational level, marital status, duration of residence, and diabetes knowledge. Individual classifiers achieved a moderate performance (accuracy: 56.7–70.0%, F1-score: 0.686–0.781). The stacking ensemble substantially outperformed individual models, achieving a 95.45% accuracy, a 100% precision, a 93.75% recall, a 0.968 F1-score, and a 0.979 AUC-ROC on the test set. **Conclusions:** Machine learning models can reliably predict diabetes. The proposed hybrid stacking model outperformed conventional classifiers in terms of the prediction performance, highlighting the benefits of ensemble learning and sophisticated resampling strategies in dealing with imbalanced medical data. It is recommended that healthcare institutions integrate AI-powered diagnostic assistance technology into clinical processes to aid in the early detection and treatment of diabetes.

## 1. Introduction

The global pandemic of diabetes mellitus, which is predicted to reach 700 million cases by 2045, represents a significant challenge to patients and medical professionals alike. The high social and economic costs of diabetes, along with its concerning prevalence rates, indicate a pressing need for more effective control and prevention measures [1].

According to World Health Organization estimates, 422 million people worldwide, mostly in low-income countries, have type 2 diabetes (T2D). Globally, the prevalence is increasing, even though early detection can save lives [2]. Periodically uncontrolled diabetes causes hyperglycemia, which damages many organs and tissues over time, including arterial capillaries and neurons [3].

According to Soni and Varma [4], people with type 2 diabetes who are homeless are five times more likely than those who are housed to report diabetes-related emergency department visits or hospital stays. Compared to people who are domiciled, the homeless population with diabetes has a higher rate of emergency room visits and hospitalizations for diabetic complications. Homeless people are particularly at risk for sexual and physical abuse; have a worse nutritional status; are less likely to take their medications as prescribed; have less reliable wound care; have less access to healthcare, which results in delayed presentations; and have higher rates of comorbidities [5].

Screening homeless people who are at risk of diabetes is essential to putting preventive measures into place on time. It is vital to identify people at risk for diabetes and involve them in the health system in areas with a lack of healthcare data and information, poor healthcare system utilization, and/or long travel times to the closest health facilities. In their capacity as community and gerontological nurses, community health workers can conduct non-invasive screening tests by visiting homes or holding community-based sessions. They can also direct high-risk individuals for diabetes to higher-level care and continue to support them [6]. Community and gerontological health nurses are well placed to provide this type of non-invasive fingertip testing. These tests provide instant results, meaning that dietary and lifestyle advice can be offered on the spot, tailored to the individual’s health needs [7].

Obesity, waist circumference measurements, and frequent consumption of foods with a high glycemic index are among the other risk factors that have been connected to the development of type 2 diabetes [8]. Type 2 diabetes mellitus is also known to be linked to demographic characteristics such as age and sex [9].

Lifestyle factors like food, exercise, and alcohol consumption have a significant impact on type 2 diabetes mellitus, which reduces life expectancy and quality of life. It is essential to diagnose and treat T2D as soon as possible to prevent patients from developing severe and perhaps fatal consequences [10].

According to Deberneh et al. [11], early disease detection allows individuals who are at risk to adopt preventative measures that will slow the disease’s progression and enhance their quality of life. Many studies using machine learning approaches have been carried out to help in the early diagnosis of T2D [10].

In addition to preventing complications, early detection of diabetes lowers the risk of acquiring other chronic conditions. It is crucial to use resources effectively to predict the disease [12]. Homeless individuals deal with food shortages, precarious housing, and disjointed services, which make traditional diabetes treatment plans impractical and increase complications [13]. Furthermore, prior research showed that individuals with a history of homelessness are more likely to have retinopathy and undergo fewer retinal screening exams [14].

Artificial intelligence is being widely used in diabetes care in four main areas: developing tools that let patients manage their conditions independently, predicting the risk for specific groups, providing clinical advice and decision support, and automatically screening for retinal disorders [15,16]. The primary goals of using machine learning techniques for predictive analytics in diabetes care are to improve clinical outcomes in the management of diabetes mellitus, optimize resource allocation, improve patient care strategies, and achieve an accurate disease diagnosis [17].

Supervised machine learning models, a subset of artificial intelligence, are useful tools for diagnosing and treating diabetes. It has been demonstrated that machine learning models are quite accurate in predicting the onset of diabetes. A person’s medical history and other risk factors serve as the foundation for these models [18]. Diabetes prevention can be achieved using a careful assessment of the patient’s sociodemographic and health conditions and implementing a personalized treatment plan that considers each patient’s specific risk factors and health conditions [19].

According to the World Health Organization [20], diabetes prediction is one of the bioinformatics domains where a variety of algorithms based on machine learning have recently been created to automatically tackle prediction or classification issues. It has been demonstrated that machine learning models are quite accurate in predicting the onset of diabetes. A person’s medical history and other risk factors serve as the foundation for these models [21,22]. Numerous predictive models for diabetes have been created by utilizing conventional statistical techniques and machine learning algorithms. Prior research has utilized logistic regression, decision trees, random forests, and gradient boosting methods to forecast diabetes based on demographic and clinical factors. Chang et al. [23] demonstrated that machine learning models integrating age, body mass index, blood pressure, and lifestyle factors can attain a robust predictive efficacy in population-based datasets. Upadhyay and Gupta [24] utilized logistic regression and ensemble learning techniques for the early identification of diabetes, demonstrating an enhanced classification accuracy through the integration of clinical and lifestyle factors. Nevertheless, the majority of current research depends on general population information and seldom addresses socially vulnerable groups, such as the homeless. Homeless populations frequently encounter distinct risk factors such as restricted healthcare access, an inconsistent diet, and a significant burden of comorbidities. As a result, predictive modeling methods particularly designed for these at-risk populations are few. Rectifying this deficiency is crucial for formulating focused screening methodologies and preventive measures.

### 1.1. Significance of the Study

Type 2 diabetes mellitus is still a rapidly expanding public health issue that has a major influence on morbidity, mortality, and healthcare resources, particularly in low-income nations like those in Africa. Regarding the number of T2DM patients worldwide, Egypt has been ranked as the ninth most populous nation. Over the past 20 years, the frequency of type 2 diabetes in Egypt has nearly tripled. It currently accounts for 15.6% of all adults between the ages of 20 and 79 [25].

The global diabetes prevalence was predicted to be 9.3% (463 million) in Egypt in 2019, 10.2% (578 million) by 2030, and 10.9% (700 million) by 2045. The incidence is higher in high-income countries (10.4%) than in low-income countries (4.0%), and the frequency is higher in urban areas (10.8%) than in rural ones (7.2%). A total of 50.1% of people with diabetes are not aware that they have the disease [26].

Diabetes mellitus is often linked to a two- to four-fold higher risk of cardiovascular and microvascular problems, some of which may exist prior to the diagnosis. Thus, early detection of pre-diabetic patients is crucial [27]. According to the disease’s current rate of growth, one in ten adults is predicted to have diabetes by 2040 [28].

In addition to having higher average glycated hemoglobin levels, which are indicative of worse diabetes control, homeless diabetics also experience a much higher rate of lower limb amputations than those who are housed [29]. When compared to 0.01% of deaths in the general population in the same age group, type 2 diabetes caused 1% of the mortality among homeless people aged 20–44 [30].

It is crucial to find predictive factors that could affect the diabetes occurrence among homeless adults. Therefore, detecting these predictive factors would help nurses provide appropriate nursing care to possibly reduce the diabetes prevalence and prevent its complications specific to the homeless. So, this study aimed to identify predictors of diabetes among homeless adults by utilizing artificial intelligence and providing recommendations for diabetes prevention. This was achieved by integrating demographic, clinical, and lifestyle variables. The goal was to enhance early detection and inform targeted preventive interventions within vulnerable communities.

### 1.2. Theoretical Framework

**Figure 1:**
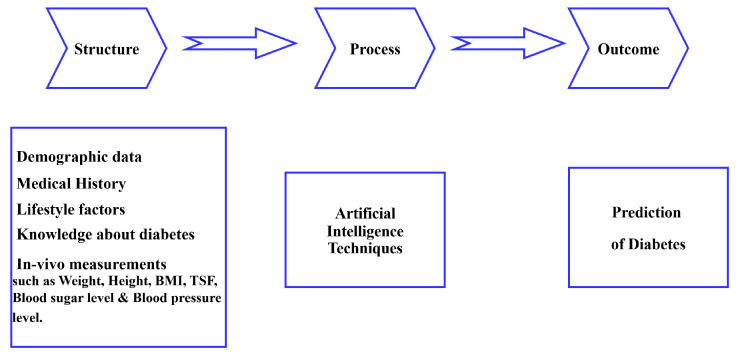
Application of Donabedian model to diabetes predictions.

This study used the Donabedian model, which assesses care in three domains: outcome, process, and structure, as illustrated in Figure 1. According to Donabedian and Hickey et al. [31,32], structure refers to all the elements that influence the setting in which care is provided, process refers to the act of giving and receiving care, and outcome refers to the impact of services on patients’ well-being.

## 2. Materials and Methods

### 2.1. Aim of the Study

The current study aimed to identify predictors of diabetes among homeless adults by utilizing artificial intelligence and providing recommendations for diabetes prevention.

### 2.2. Research Hypothesis

To achieve the aim of this study, the following research hypothesis was formulated:

**Hypothesis** **1.** *Artificial intelligence-based predictive models can accurately identify the risk of diabetes among homeless adults using demographic, clinical, and lifestyle-related variables.*

This study explicitly examined the implementation of machine learning techniques within a socially vulnerable group (homeless adults), a topic that has been infrequently investigated in prior diabetes prediction research, by incorporating clinical, lifestyle, and behavioral variables within this demographic.

### 2.3. Research Design

The current study used a case-control study design. A group of cases with the outcome of interest is the starting point for a case-control study. The researcher then attempts to create a second set of people, known as the controls, who are comparable to the case individuals but do not experience the desired result. After examining past data, the researcher determines whether any exposures are more prevalent in the cases than in the controls. If the exposure is more prevalent in the cases than in the controls, the researcher can hypothesize that it is connected to the outcome of interest [33]. The cases were homeless adults diagnosed with type 2 diabetes mellitus, defined by a fasting blood sugar (FBS) ≥ 126 mg/dL or a prior medical diagnosis. The controls were homeless adults without diabetes, confirmed by an FBS < 126 mg/dL and no history of the disease.

The study employed a case-control sample design, with the main aim of investigating the possibility of creating predictive models for diabetes in a hard-to-reach community, rather than estimating exposure–outcome relationships. The case-control approach facilitated the effective gathering of clinical and lifestyle data from a restricted cohort of homeless individuals within the institutional environment. The dataset thus constitutes a structured sample rather than the actual population prevalence of diabetes. Thus, model performance measurements are to be regarded as markers of internal prediction discrimination rather than as estimations of the actual screening efficacy in real-world scenarios. This design methodology has been employed in exploratory machine learning research where access to representative cohort data is constrained.

### 2.4. Setting

The study was conducted at the Ma’ana Rescue Human Foundation. The foundation operates two primary branches: one for women and another for men in Giza Governorate, Egypt. It was established in response to the growing number of homeless individuals across Egypt’s streets and governorates. The founders were deeply concerned by the alarming increase in this vulnerable population, who, without shelter, food, medical care, or hygiene, experience severe physical and psychological deterioration. Many suffer from untreated illnesses, leading to infections and the necessity of limb amputations in some cases. The foundation aims to be the first nationwide initiative to create shelters for the homeless across all Egyptian governorates, providing them with essential living conditions such as safe accommodation, food, clothing, medical treatment, and even surgeries, all free of charge. The foundation’s services, including housing, healthcare, and personal hygiene, are available 24 h a day, with a range of activities like sports and job opportunities for those capable of working. Additionally, the institution strives to reintegrate homeless individuals into society by addressing their personal issues, modifying behaviors, and ensuring that all residents live with dignity and respect.

### 2.5. Sample

The total number of homeless was found to be approximately 170 people. Slovin’s formula was used to calculate the sample size for the study and is expressed as n = N/(1 + Ne^2^), where n = sample size, N = total population size, and e = margin of error, typically 0.05 for a 95% confidence level [34].

$$n=\frac{170}{1+170\times 0.05^{2}}\approx 120.$$

A purposive sample of 150 homeless adults was included in this study according to the following inclusion criteria: aged 18 years and older, of either sex, able to communicate, and free from mental problems.

Utilizing Slovene’s technique, the minimum requisite sample size was calculated to be roughly 120 participants to attain an acceptable margin of error at a 95% confidence level. Nonetheless, due to the limited and reachable population at the research location and the exploratory characteristics of predictive modeling, a bigger sample was deliberately gathered. Consequently, 150 homeless people were incorporated into the final analysis to augment the robustness and stability of the statistical comparisons and machine learning models, mitigate potential sample bias, and boost the reliability of model performance estimations.

The sample size adhered to viable population bounds and surpassed the predicted minimum need, which is methodologically sound for predictive analytics in small and difficult-to-access populations. The present study comprised 99 cases and 51 controls, reflecting the actual distribution of diabetes within the closed homeless population under investigation. Participant recruitment was based on eligibility within a single institutional setting rather than on artificial matching or predetermined case-control ratios.

Given that the primary objective of the study was predictive modeling rather than estimation of exposure–outcome associations, strict adherence to conventional matching ratios was not required. The observed class imbalance represents the true prevalence within the study population. To address this imbalance methodologically while maintaining an unbiased model evaluation, the synthetic minority over-sampling technique (SMOTE) was applied exclusively to the training dataset.

### 2.6. Tools for Data Collection

After reviewing the related literature, the following two tools were developed by the researchers and used for data collection in this study:

#### 2.6.1. First Tool

A structured interviewing questionnaire for homeless adults, which consists of four parts:

Part I: demographic data (6 questions); Part II: medical history (5 questions); Part III: lifestyle factors (15 questions); and Part IV: knowledge about diabetes (7 questions).

Lifestyle scoring system:

The questionnaire includes 15 questions with 3 items: yes, sometimes, and no, where yes scored 3 points, sometimes scored 2 points, and no scored 1 point. The total lifestyle scores ranged from 15 to 45 points and were classified as the following: 15–24 points indicates an unhealthy lifestyle, 25–34 points indicates a moderate lifestyle, and 35–45 points indicates a healthy lifestyle [35].

Knowledge scoring system:

The questionnaire consisted of multiple-choice questions. A correct, complete answer scored 3; a correct, incomplete answer scored 2; and an incorrect, or “I don’t know,” response scored 1. The total scores of knowledge were computed by summing the correct responses to all questions. The total knowledge scores ranged from 7 to 21 points. The level of knowledge was classified into three levels: 7–11 points (less than 50%) indicates low knowledge, 12–16 points (50–75%) indicates moderate knowledge, and 17–21 points (more than 75%) indicates high knowledge [36].

#### 2.6.2. Second Tool

Physiological measurements, such as the weight, height, body mass index (BMI), waist circumference, triceps skin fold thickness (TSF), blood sugar level (BSL), and blood pressure level. The body mass index was classified according to the World Health Organization [37]: <18.5 kg/m^2^ indicates underweight, 18.5–24.9 kg/m^2^ indicates a normal weight, 25–29.9 kg/m^2^ indicates overweight, and ≥30.00 kg/m^2^ indicates obese. Central obesity was defined as a waist circumference ≥ 90 cm in men or ≥85 cm in women [38]. A normal triceps skin fold thickness for women is 15–25 mm, and for men, it is 10–15 mm [39]. Type 2 diabetes mellitus was diagnosed by a fasting blood sugar level (FBS) of 126 mg/dL or higher [40]. Hypertension was defined as systolic blood pressure (SBP)/diastolic blood pressure (DBP) ≥ 140/90 mm Hg or a history of hypertension [41].

Tool Validity and Reliability:

A panel of three experts in the fields of computers and artificial intelligence, community health nursing, and geriatric health nursing reviewed the developed tools to determine the validity of their content. In compliance with the experts’ judgments on the appropriateness of the contents, modifications were carried out. The Cronbach’s alpha reliability test was used; the reliability of the first tool was 0.804 and the reliability of the second tool was 0.716. The lifestyle questionnaire used in this study was adapted from a previously validated 15-item lifestyle screening tool developed by Hwang [35] for community-dwelling adults. The original instrument demonstrated satisfactory psychometric properties, including an acceptable content validity and internal consistency reliability. For the current study, minor contextual modifications were made to ensure cultural appropriateness and relevance to the homeless population, without altering the core conceptual domains.

### 2.7. Procedure

Before the study began, the researchers explained its purpose to each participant to gain their cooperation. Interviewing the participants was carried out inside their rooms/the institutions’ garden. Before distribution of the questionnaire, the researchers informed each participant about the confidentiality of the collected data and their right to withdraw from the study at any time, and then the questionnaire was distributed. The researchers were present with each participant while they filled out the questionnaire to ensure that individual responses were provided by everyone. The questionnaire was filled in the presence of a responsible person at the institution. The time spent to fill the questionnaire ranged between 15 and 20 min, and the researchers met the participants twice per week from 9 a.m. to 2 p.m.

Measurements were taken by the researchers in the participant’s room/the institution’s garden. The weight, height, and body mass index were measured using a digital balance scale and tape; the height and weight were measured without shoes and in light clothes, and the BMI (kg/m^2^) was calculated as the weight in kilograms divided by the height in meters squared. The waist circumference was taken using tape and measured at the midpoint between the lowest rib and the iliac crest on the mid-axillary line. The triceps skinfold thickness was taken using tape and a skinfold caliper by grasping a fold of skin and subcutaneous adipose tissue approximately 2.0 cm above the mid-arm circumference mark. The blood sugar level was measured by using an electronic blood glucose meter and test strip. The blood pressure was measured twice by a mercury sphygmomanometer, and the average was calculated.

Prediction of diabetes using artificial intelligence among homeless adults was done through the following steps: The demographic data, medical history, lifestyle factors, knowledge about diabetes, and physiological measurements were obtained. Then, the subjects were divided into two groups: one group with diabetes, and the other without diabetes. After that, the artificial intelligence techniques were used by the python program to select the strongest predictors from all factors to determine the predictors affecting the occurrence of diabetes for the participants.

Finally, the data collected from the participants were divided into two parts (training samples and test samples) so that about 80% of the data were entered for the artificial intelligence techniques used for training and 20% were used for testing the techniques after their design. Thus, the number of correct cases collected from the data used in the test was divided by all cases multiplied by 100 to find out the success rate of the method, so that it reached a success rate greater than or equal to 90%. Structured suggested recommendations were carried out for the participants at the end of the study through oral instructions as a method of teaching supported with a booklet containing all the information and skills related to the prevention of diabetes mellitus.

### 2.8. Data Preprocessing, Model Development, and Validation

#### 2.8.1. Data Preprocessing

The data were assessed for completeness and consistency prior to model development. Missing values were evaluated in terms of their pattern and proportion. Variables exhibiting more than 20% missing data were excluded from the analysis. For the remaining variables, missing continuous values were imputed using the median, whereas categorical variables were imputed using the mode to minimize distributional distortion.

Categorical variables (e.g., gender, smoking status, and educational attainment) were encoded using one-hot encoding. Continuous variables (e.g., age, body mass index, and fasting blood glucose) were standardized using *z*-score normalization to ensure uniform scaling across predictors and to enhance the algorithm performance.

Feature selection was performed using a combination of a correlation analysis and recursive feature elimination (RFE) to reduce multicollinearity and retain the most informative predictors for model training. Feature selection was implemented, notwithstanding the limited dataset, to enhance model generalization and mitigate the risk of overfitting. When the number of predictors approaches the number of observations, machine learning algorithms may capture noise rather than meaningful patterns. Therefore, feature-selection techniques, including recursive feature elimination, univariate analyses, and correlation analyses, were employed to identify the most relevant predictors while removing redundant or highly correlated variables. This process improves model interpretability and may enhance prediction stability when working with limited datasets.

#### 2.8.2. Missing Data Management

The dataset exhibited negligible missing values (<3% for any variable). Missing data were addressed using multiple imputation by chained equations (MICE), which generates plausible values based on correlations with other variables. Sensitivity analyses comparing a complete-case analysis with imputed datasets revealed no significant differences in the model performance, thereby confirming the robustness of the imputation strategy.

#### 2.8.3. Model Development and Hyperparameter Tuning

Several machine learning algorithms were evaluated, including random forest, support vector machine (SVM), K-nearest neighbors (KNN), and gradient boosting classifiers. Hyperparameter tuning was conducted using grid search combined with 5-fold cross-validation on the training dataset to identify optimal parameter settings and reduce overfitting.

The dataset was randomly split into training (80%) and testing (20%) subsets. The model performance was internally validated using cross-validation within the training set prior to the final evaluation on the independent test set.

#### 2.8.4. Model Evaluation and Calibration

The model performance was assessed using the accuracy, precision, recall, F1-score, and area under the receiver operating characteristic curve (AUC-ROC). The calibration performance was evaluated using calibration plots and the Brier score to measure the agreement between the predicted probabilities and the observed outcomes.

Continuous predictors such as the age, BMI, waist circumference, and blood pressure were retained as standardized continuous variables rather than categorized where possible to preserve statistical information and avoid loss of predictive power.

#### 2.8.5. Reporting Standards

The development and reporting of the prediction model followed the TRIPOD-AI guidelines to ensure transparency, reproducibility, and comprehensive methodological reporting.

### 2.9. Proposed Model

**Figure 2:**
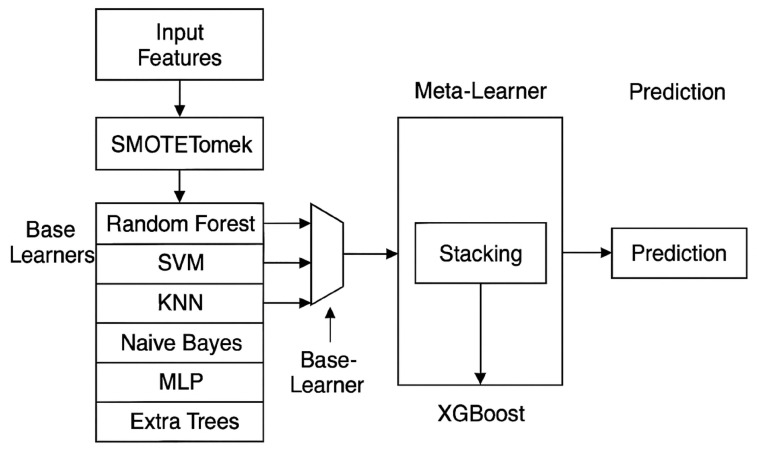
Architecture of the optimized hybrid meta-learning classifier for diabetes predictions among homeless adults.

As shown in Figure 2, six well-known machine learning algorithms were used to set a standard for diabetes classification: logistic regression (LR), a support vector machine (SVM), random forest (RF), a decision tree (DT), K-nearest neighbors (KNN), and gradient boosting (GB). These models were chosen based on their proven effectiveness in biomedical classification tasks and their ability to capture a wide range of data patterns, from linear to highly nonlinear correlations. All the models were trained on standardized features and tested on an unseen 20% test set without using data-balancing techniques.

Each algorithm contributes a distinct modeling approach to the categorization task. Logistic regression is a simple and easy-to-understand method, while a SVM, especially with a radial basis function kernel, is great at handling complicated relationships in data with many dimensions. Tree-based approaches, such as decision trees and random forests, offer interpretable decision-making structures that are resistant to noise and feature interactions. KNN uses a method based on measuring distances between data points, while gradient boosting builds strong prediction models step by step by fixing mistakes from earlier models, leading to very accurate predictions when adjusted correctly.

Given the limited sample size (n = 150) and the class imbalance between diabetic and non-diabetic subjects, the synthetic minority over-sampling technique (SMOTE) was utilized in the data preparation phase. SMOTE was employed to synthetically create additional samples for the minority class by interpolating between existing examples in the feature space, thus enhancing the class distribution and model learning efficacy. To avert information leakage and guarantee an impartial evaluation, oversampling was conducted exclusively on the training dataset, while the test dataset remained unaltered and was utilized solely for a performance assessment. The utilization of SMOTE is well recognized in medical machine learning research, especially when addressing constrained and imbalanced datasets, and it enhances the model stability and prediction efficacy.

The hybrid stacking ensemble was executed as detailed below:

1. Meta -feature generation: The meta-learner was trained utilizing anticipated class probabilities, rather than definitive class labels, produced by each base learner.

2. Out-of-fold predictions: To avert information leaking, k-fold cross-validation (k = 5) was utilized on the training set. The out-of-fold predicted probabilities from each base model were used as inputs for training the XGBoost meta-learner.

3. Fina l evaluation: The comprehensive stacking model was assessed once on an unobserved 20% test set, which was excluded from both the training and validation phases.

These measures guaranteed that the meta-learner was not privy to the ground-truth labels from the data utilized to create its input features, thereby mitigating the likelihood of overfitting.

### 2.10. Statistical Analysis

The collected data were scored, tabulated, computed, and analyzed using the Statistical Package for the Social Sciences program, version 26. Descriptive and inferential statistics were used to present the collected data. Qualitative variables were displayed as numbers and percentages. Quantitative variables were presented as the mean ± standard deviation (SD). Relationships between the variables were determined by using Pearson’s chi-square test to calculate the association between diabetes and the study’s independent variables. For prediction of diabetes, Python 3.13 was used. Python 3.13 is highly effective for statistical analyses in predictive modeling due to its improved performance and new features that assist in handling complex computations. It allows analysts to build models that can learn from historical data and provide valuable forecasts based on artificial intelligence with a greater accuracy and efficiency.

#### Fundamental Multivariable Logistic Regression

A multivariable logistic regression model was developed to supplement the machine learning models and establish an interpretable statistical baseline, utilizing the predictors identified during the feature-selection phase. Adjusted odds ratios (AORs) with 95% confidence intervals (CIs) were calculated to quantify the strength of association between the predictors and the diabetes status. This approach allows for comparison between traditional statistical modeling and machine learning techniques, while improving the interpretability of the identified predictors.

## 3. Results

### 3.1. Demographic and Medical History of Homeless Adults

The study included 150 homeless adults, with 99 diagnosed with diabetes and 51 without diabetes. Table 1 presents the demographic and medical history characteristics of the study participants, comparing diabetic and non-diabetic groups.

Table 1 illustrates that 62.6% of the diabetic homeless were aged 60 years or more, with a mean age of 58.62 ± 9.19 years old, and they were male; 74.8% were from urban areas. Regarding the educational level, 35.3% of them could not read and write, and 33.3% were single. In relation to the duration of residence in the current location, 59.6% were there for less than 3 years, with a mean of 2.71 ± 1.47 years; 40.4% were suffering from hypertension; 44.4% did not take medication regularly; and 14.1% were diagnosed with high cholesterol. A total of 79.8% did not have health insurance, and 64.6% reported that the organization did not conduct regular medical check-ups. A highly significant association was found among the case’s age, their marital status, the presence of other diseases, the use of medication regularly, and a previous diagnosis with high cholesterol.

### 3.2. Lifestyle and Knowledge of Homeless Adults

Table 2 shows that non-diabetic homeless people reported eating snacks regularly at a higher rate (33.3%) compared to diabetic individuals (16.2%). Regular physical activity was more commonly reported among non-diabetics (31.4%) compared to diabetics (16.1%), whereas diabetics more frequently selected “sometimes”. A total of 9.1% of diabetics reported smoking compared to 3.9% of non-diabetics.

Table 3 clarifies that 82.8% and 57.6% of homeless diabetics had incorrect knowledge about the definition and tests used to detect diabetes, respectively, while 17.6% of non-diabetic people had correct and complete knowledge about the definition of diabetes.

### 3.3. Physiological Measurements

Table 4 shows that 40.4% of diabetic homeless had a body mass index of 25–29.9 kg/m^2^, and 37.3% had ≥30.00 kg/m^2^ with a mean of 27.78 ± 4.06 kg/m^2^. Regarding waist circumference, 80.8% were ≥85 cm for women and ≥90 cm for men, with a mean of 99.3 ± 27.57 cm. In relation to the triceps skin fold thickness, 67.6% were >15 for men and >25 for women, with a mean of 27.7 ± 19.03. In terms of the blood pressure level, 85.9% were more than 140/90 mm Hg, with a mean of 131/82 ± 52/14 mm Hg. A highly significant association was found between the cases’ body mass index and their waist circumference and blood pressure level.

**Figure 3:**
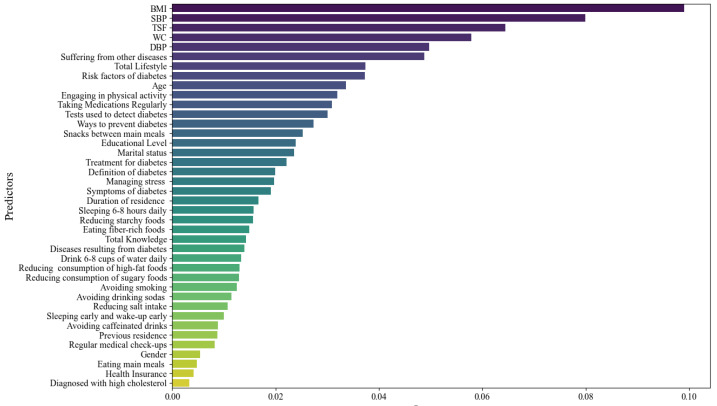
The most important predictors of diabetes using the proposed model.

As shown in Figure 3, the 13 most important variables for predicting diabetes include the BMI, SBP, TSF, waist circumference, lifestyle factors, presence of other diseases, DBP, age, regular medication use, educational level, marital status, duration of residence, and knowledge about diabetes.

### 3.4. Machine Learning Model Performance

The evaluation metrics were calculated based on the confusion matrix derived from the test dataset. The accuracy, precision, recall, and F1-score were computed using standard classification performance formulas to ensure consistency among the reported metrics.

As shown in Table 5, the hybrid model surpassed all baseline models in all evaluation parameters, with an F1-score of 0.9677 and an AUC of 0.9792, demonstrating a strong precision and generalization ability.

Hyperparameter Optimization: All models were subjected to hyperparameter optimization by a grid search employing 5-fold cross-validation on the training dataset as shown in Table 6. Tuning was conducted individually for each algorithm to guarantee an optimal performance.

Five-fold cross-validation was performed on the training dataset to acquire more reliable estimates of the model performance. The training data were partitioned into five subsets, with four subsets designated for model training and one subset allocated for validation. The procedure was executed five times, and the average performance metrics were computed.

All hyperparameter optimizations were performed alone on the training data to avert information leaking. The final models were retrained on the complete training set utilizing optimal parameters prior to assessment on the reserved test set.

The influence of feature selection was assessed by comparing the model performance prior to and during the feature-reduction process, as seen in Table 7. The findings demonstrate that the majority of models exhibited an enhanced predictive performance following the selection of the most pertinent predictors. The inclusion of all accessible variables led to a somewhat diminished performance, probably due to redundant or less informative features that augmented the model complexity.

Subsequent to the implementation of feature selection, the models exhibited an enhanced classification performance and stability. The hybrid meta-learning model exhibited the greatest significant enhancement, with its accuracy rising from 0.82 to 0.95 and its AUC increasing from 0.87 to 0.98. These findings underscore the significance of feature selection in enhancing model generalization and mitigating potential overfitting, particularly when dealing with relatively small datasets.

**Evaluation metrics and their descriptions:**

**Accuracy** determines the total correctness of forecasts across all classes. Accuracy is calculated as (TP + TN)/(TP + FP + TN + FN), where TP stands for true positive, TN for true negative, FP for false positive, and FN for false negative.

**Precision (P)** denotes the percentage of successfully predicted positive cases among all predicted positives. Precision = TP/(TP + FP).

**Recall (R)** assesses the ability to correctly identify all genuine positive examples. Recall equals TP divided by (TP plus FN).

**The F1-score** achieves a balance of precision and recall, which is particularly effective in imbalanced datasets. The F1-score is calculated as (2 × P × R)/(P+R).

**The AUC-ROC** measures the model’s ability to discriminate between classes at various thresholds. The AUC-ROC does not have a single formula because it sums the performance across all thresholds.

## 4. Discussion

Homeless individuals are subjected to many challenging circumstances, especially accessing healthcare and maintaining proper health. They are particularly vulnerable to diabetes due to factors such as an increase in age and lack of proper diet and exercise [42]. Artificial intelligence is enhancing the prevention, diagnosis, and management of diabetes. The current study revealed that more than half of diabetic homeless were aged 60 years or more with a mean age of 58.62 ± 9.19 years old, and they were male; more than two-thirds were from urban areas, and one-third of them were single. In relation to duration of residence in the current location, about sixty percent were there for less than 3 years, with a mean of 2.71 ± 1.47 years. These results were consistent with those of a study done by Konrady and Talarska [43]; they discovered that 75.4% were male, the average age was 55.9 ± 12.1 years, and 68.4% of them stayed at home for less than 3 years. Similarly, the study conducted by Zhang et al. [44] found that 25.3% of the participants were single, and most of them came from urban areas.

The current study showed that two-fifths were suffering from hypertension, and more than two-fifths did not take medication regularly, while less than one-fifth were diagnosed with high cholesterol. These findings were corroborated by Asgary et al. [45]; they found that a significant number had high blood pressure and hyperlipidemia. In a comparable way, Sharan et al. [46] found that 66.6% of the participants had hypertension, while 49.9% had hyperlipidemia, and they were significantly more likely to have inadequately managed hypertension. This may be due to less adherence to medications or diet due to limited access to healthy food choices or exercise chances during homelessness.

The majority of homeless adults did not have health insurance, and more than two-thirds reported that the organization did not conduct regular medical check-ups. On the contrary, a study conducted by Asgary et al. [45] found that 90% of homeless people have health insurance. Also, Bommer et al. [47] revealed that most participants had health insurance. Geographic and socioeconomic variables could account for these discrepancies.

The findings of the current study indicated that approximately one-third of the non-diabetic homeless individuals reported regularly eating snacks, and a similar proportion reported engaging in regular physical activity. This finding matched with the results of the study done by Jamir and Sudharshini [48], which revealed that 27.6% of the participants had a habit of smoking daily and 1.5% had a habit of doing moderate exercise daily. And they recommended that awareness of lifestyle risk factors among the homeless population is essential to ensure their healthy living.

At the same line, Zhang et al. [44] showed that individuals with diabetes are significantly more likely to be smokers, engage in less physical activity, have longer daily sitting times, consume more processed meat, and have a lower milk intake. SumaLata and Joshitha [49] added that diabetes is caused by overweight or obesity, which is due to a lack of physical activity and causes resistance to insulin. This further causes one of the dangerous risk factors for cardiovascular diseases. Type 2 diabetes is due to lifestyle, physical activity, and diet.

The current study revealed that most homeless individuals with diabetes had incorrect knowledge regarding the definition of diabetes, and more than half had incorrect knowledge about the diagnostic tests, whereas less than one-fifth of non-diabetic individuals demonstrated correct and complete knowledge of the disease definition. This result was consistent with the findings of a study conducted by Maury et al. [50]; they found that homeless people had low knowledge about the causes and treatment of diabetes, proper diet and exercise, symptoms of hyperglycemia and hypoglycemia, and how to treat these conditions. Along the same lines, Alemayehu et al. [51] revealed that the level of knowledge regarding diabetes mellitus was low among participants, which indicates a need for health education and early identification.

The results of the current study showed that two-fifths of the participants were overweight, and more than one-third were obese. These results were contrary to the results of the study conducted by Bawah et al. [52], who found that 33.1% were overweight and 26.1% were obese. The difference between the two studies may be attributed to variations in socioeconomic conditions, nutritional patterns, and healthcare accessibility among countries.

The results of the current study revealed that most participants had hypertension. This result was in line with the study conducted by Gu et al. [53], who found that 65% had elevated blood pressure. The present results, however, contradicted those of a study by Asgary et al. [45], who found that 31.3% had uncontrolled hypertension (≥140/90). This variation could be explained by the fact that the current study assessed homeless adults regardless of their prior diagnosis or treatment. Furthermore, there may have been differences in the healthcare systems, access to medication, lifestyle factors, and social support between the two settings.

The results of the current study indicated that most participants had central obesity, with a mean of 99.3 ± 27.57 cm. A significant association was found between the cases’ waist circumference and blood pressure level. These results were congruent with the study done by Chen et al. [54], who found that 54% of the participants had central obesity and a significant association was found with their waist circumference and blood pressure level. The consistency between both studies reinforces the well-established link between abdominal obesity and cardiovascular risks, particularly hypertension. Waist circumference is a reliable indicator of visceral fat, which can elevate blood pressure. Also, these results were matched with the result of a study done by Gu et al. [53], who found that 54% of participants had central obesity with a mean of 90.0 ± 12.7 cm.

The results of the current study depicted that more than half of the participants had high fat. This result is in harmony with a study by Alkhatib et al. and Gina et al. [55,56]; they mentioned that most of the participants had high fat, and skin thickness may lead to diabetes. This result was contrary to the results of the study conducted by Langnäse and Müller [57], who found that 22.7% of the participants were obese and had high fat. This discrepancy may reflect geographic and socioeconomic differences between the two populations.

The results of the current study demonstrated that the most significant predictors of diabetes include the BMI, SBP, TSF, waist circumference, lifestyle factors, presence of other diseases, DBP, age, regular medication use, educational level, marital status, duration of residence, and knowledge about diabetes. The hybrid model markedly surpassed the individual classifiers, attaining an accuracy of 95.45%, an F1-score of 0.967, and an AUC of 0.979.

These results supported the research conducted by SumaLata and Joshitha [49] that incorporated clinical, demographic, and lifestyle data to predict diabetes using machine learning algorithms. The feature-selection process identified significant predictors, such as the blood pressure, body mass index, glucose levels, and family history of diabetes. The research outcomes provide valuable perspectives on the optimal amalgamation of AI techniques for diabetes predictions.

Ojurongbe et al. [58] showed that several clinical symptoms, demographic characteristics, and patients’ diabetes knowledge were significantly correlated with the ability to predict when the condition would manifest. Therefore, it is crucial to raise awareness of diabetes risk factors, encourage healthy lifestyles, and emphasize the importance of an early diagnosis and treatment to reduce the prevalence of diabetes and its complications.

The predictive model, based on multivariable penalized logistic regression, achieved an AUC of 99% for the training set and 94% for the test set.

Upadhyay and Gupta [24] created a machine learning model for the early detection of diabetes using a methodical approach. They assessed models using a variety of metrics to verify the accuracy, precision, F1-score, recall, and ROC curve. When compared to other classification models in this evaluation, logistic regression performed best, with an accuracy of 82% and an ROC curve of 87%. Glucose and the body mass index are two indicators directly associated with an increase in diabetes. Ensemble models can outperform individual classifiers in diabetes prediction tasks. In comparison, the stacking ensemble developed in this study achieved a high predictive performance while specifically focusing on a homeless population, which has been rarely represented in the existing diabetes prediction research.

A study done by Chang [23] used various machine learning methods to forecast a diabetes diagnosis. Type 2 diabetes was predicted using five machine learning classifiers: Gaussian naive Bayes, random forest, K-nearest neighbors, a decision tree, and logistic regression. It was found that the primary risk factors for diabetes are income, walking difficulties, general health, physical health, blood pressure, cholesterol, body mass index, and age. The predictive performance of the proposed stacking model was comparable to the results reported in previous diabetes prediction studies. Machine learning models achieved accuracies between 85% and 92% using demographic and clinical predictors.

### 4.1. Interpretation of Predictors

The 13 identified predictors align with established diabetes risk factors, supporting biological plausibility. However, alternative explanations for these associations deserve consideration.

#### 4.1.1. Age and Diabetes

While age is a well-established risk factor, its strong predictive weight in our model may partially reflect a longer undiagnosed diabetes duration due to limited healthcare access, rather than purely incident risk.

#### 4.1.2. BMI and Central Obesity

These anthropometric measures showed strong predictive value, consistent with the obesity–diabetes link. However, in homeless populations, food insecurity and undernutrition coexist with obesity, potentially creating complex relationships not captured by the BMI alone.

#### 4.1.3. Lifestyle Factors

The moderate-to-unhealthy lifestyle patterns among diabetic cases could represent either causal factors or consequences of diabetes (e.g., fatigue reducing physical activity). The cross-sectional design cannot distinguish directionality.

#### 4.1.4. Diabetes Knowledge

A lower knowledge among diabetic cases may reflect limited health education access, but could also result from cognitive effects of chronic hyperglycemia or greater psychological distress. Longitudinal studies are needed to establish temporal relationships.

#### 4.1.5. Medication Adherence

While included as a predictor, medication adherence in diabetic cases likely reflects disease management rather than pre-disease risk. Its inclusion in prediction models for undiagnosed diabetes requires careful interpretation.

### 4.2. Strengths and Limitations

This study has various strengths, including the unique use of artificial intelligence to predict diabetes in a particularly vulnerable group. In addition, the study collected detailed data through structured interviews and physiological measurements, which increased the dataset’s richness. However, the study has limitations, including a small sample size obtained through purposive sampling, which restricts its generalizability. The study employed purposive sampling from a single foundation serving homeless adults, which may limit its representativeness and external validity. However, this foundation is the largest institution providing services to homeless individuals in the region, encompassing a substantial and heterogeneous segment of this population. Nevertheless, as the data were collected from one institutional setting, the generalizability of the findings to other contexts should be interpreted with caution. The relatively small sample size increases the risk of overfitting and limits the generalizability. Although the foundation serves a heterogeneous population, the findings should be interpreted cautiously. The AI model is presented as a feasibility demonstration rather than a clinical decision-support tool. To enhance the robustness, strict train–test separation was applied. K-fold cross-validation within a stacking framework was also implemented. SMOTE was used exclusively on the training dataset to reduce class imbalance and optimistic bias. Future research should include larger, multi-center samples and external validation to confirm and generalize the findings.

The dataset, obtained through a case-control sampling strategy, does not accurately represent the actual prevalence of diabetes among homeless adults, resulting in potentially optimistic predictive performance metrics that necessitate external validation with population-based datasets.

The exceptionally high predictive performance of the stacking model must be evaluated with caution due to the relatively short test dataset (n = 30), which may have resulted in overly optimistic performance estimations.

### 4.3. Implications for Nursing Practice

This study’s findings have important implications, particularly in terms of increasing early detection and preventative care for diabetes among vulnerable populations such as the homeless. Nurses can improve the diabetes screening accuracy and efficiency by incorporating AI-powered diagnostic technologies into clinical settings. This method enables early detection of high-risk patients, allowing for prompt actions that may lessen the burden of complications and healthcare costs.

## 5. Conclusions

The study results indicate that artificial intelligence techniques can reliably predict diabetes. Integrating AI into nursing assessments and care planning enhances the ability to identify high-risk individuals early, thereby improving the health outcomes. The proposed hybrid stacking model outperformed conventional classifiers in terms of its prediction performance, highlighting the benefits of ensemble learning and sophisticated resampling strategies in dealing with imbalanced medical data. “This study makes several original contributions to the fields of diabetes research, artificial intelligence in healthcare, and community health nursing. It represents the first application of ensemble machine learning methods specifically for diabetes prediction in homeless adults, demonstrating that sophisticated AI techniques can achieve exceptional accuracy (95.45%) even with relatively small samples when appropriate resampling and cross-validation strategies are employed. The identification of 13 key predictors—including several psychosocial and contextual factors rarely included in conventional diabetes risk models—provides a novel framework for understanding diabetes etiology in the context of homelessness. Furthermore, the successful implementation of a stacking ensemble with six diverse base learners offers a methodological contribution that can be applied to other prediction tasks with imbalanced medical data. Finally, the integration of AI predictions with structured nursing recommendations provides a practical pathway for translating computational advances into improved care for underserved populations.

## 6. Recommendations

Based on the findings of this study, the following recommendations are suggested:

1. Lifestyle modifications include a healthy diet, increased physical activity, and encouraged weight loss for overweight or obese individuals.
2. Healthcare institutions should incorporate AI-driven diagnostic support technologies into clinical workflows to facilitate the early detection and treatment of diabetes.
3. Ongoing investigation into interpretable AI methodologies are advocated to enhance implementation in practical medical environments.
4. Education and training for healthcare professionals are crucial for effectively integrating AI into clinical practice and communicating its benefits and limitations to patients.
5. Regular and ongoing early detection and appropriate treatment of pre-diabetics should be performed by authorized personnel in each country.
6. Awareness programs should be implemented on a large scale after identifying the appropriate means of message spread among pre-diabetic homeless adults.

## Figures and Tables

**Table 1:** Percentage distribution of demographic data and medical history of homeless adults (n = 150).

Variables	Diabetic (n = 99)	Non-Diabetic (n = 51)	Chi-Square	df	*p*-Value
**Age (years):**					
18 to less than 40	2 (2.1%)	5 (9.8%)	10.520	6	0.010 **
40 to less than 50	21 (21.2%)	7 (13.7%)			
50 to less than 60	14 (14.1%)	11 (21.6%)			
60 or more	62 (62.6%)	28 (54.9%)			
Mean ± SD	58.62 ± 9.19	56.57 ± 11.51			
**Gender:**					
Male	62 (62.6%)	31 (60.8%)	0.129	2	0.937
Female	37 (37.4%)	20 (39.2%)			
**Previous residence:**					
Urban	74 (74.8%)	37 (72.5%)	1.283	2	0.526
Rural	25 (25.2%)	14 (27.5%)			
**Educational level:**					
Cannot read and write	35 (35.3%)	18 (35.3%)	7.946	10	0.634
Can read and write	29 (29.3%)	18 (35.3%)			
Primary education	9 (9.1%)	6 (11.8%)			
Preparatory education	20 (20.2%)	5 (9.8%)			
Secondary education	6 (6.1%)	4 (7.8%)			
**Marital status:**					
Single	33 (33.3%)	26 (50.9%)	21.681	6	0.001 **
Married	21 (21.2%)	6 (11.8%)			
Divorced	22 (22.2%)	15 (29.4%)			
Widowed	23 (23.3%)	4 (7.9%)			
**Duration of residence:**					
Fewer than 3 years	59 (59.6%)	24 (47.1%)	12.724	10	0.240
3-6 years	40 (40.4%)	27 (52.9%)			
Mean ± SD	2.71 ± 1.47	3.09 ± 1.5			
**Suffering from other diseases:**					
Hypertension	40 (40.4%)	8 (15.7%)	72.957	22	<0.001 **
Heart diseases	8 (8.1%)	3 (5.9%)			
Arthritis	4 (4.1%)	4 (7.9%)			
Osteoporosis	9 (9.1%)	5 (9.8%)			
None	38 (38.3%)	31 (60.7%)			
**Take medications regularly:**					
Yes	55 (55.6%)	16 (31.4%)	69.561	14	<0.001 **
No	44 (44.4%)	35 (68.6%)			
**Diagnosed with high cholesterol:**					
Yes	14 (14.1%)	3 (5.9%)	13.496	2	0.001 **
No	85 (85.9%)	48 (94.1%)			
**Have health insurance:**					
Yes	20 (20.2%)	9 (17.7%)	2.112	2	0.348
No	79 (79.8%)	42 (82.3%)			
**Organization conducts regular medical check-ups:**					
Yes	35 (35.4%)	24 (47.1%)	1.977	2	0.372
No	64 (64.6%)	27 (52.9%)			

** highly significant at < 0.01.

**Table 2:** Percentage distribution of lifestyles of homeless adults (n = 150).

Variables	Diabetic (n = 99)			Non-Diabetic (n = 51)		
	Yes	No	Sometimes	Yes	No	Sometimes
Eat main meals	4 (4%)	4 (4%)	91 (92%)	3 (5.9%)	2 (3.9%)	46 (90.2%)
Eat snacks	16 (16.2%)	35 (35.4%)	48 (48.4%)	17 (33.3%)	19 (37.3%)	15 (29.4%)
Eat fiber-rich foods	30 (30.3%)	12 (12.2%)	57 (57.5%)	26 (50.9%)	6 (11.8%)	19 (37.3%)
Reduce high-fat foods	22 (22.2%)	25 (25.3%)	52 (52.5%)	10 (19.6%)	12 (23.5%)	29 (56.9%)
Reduce sugary foods	29 (29.3%)	18 (18.2%)	52 (52.5%)	15 (29.4%)	8 (15.7%)	28 (54.9%)
Reduce starchy foods	25 (25.3%)	21 (21.2%)	53 (53.5%)	15 (29.4%)	11 (21.6%)	25 (49.0%)
Reduce salt intake	15 (15.2%)	25 (25.3%)	59 (59.5%)	8 (15.7%)	10 (19.6%)	33 (64.7%)
Drink 6–8 cups of water daily	20 (20.2%)	33 (33.3%)	46 (46.5%)	9 (17.6%)	23 (45.1%)	19 (37.3%)
Avoid sodas	19 (19.2%)	31 (31.3%)	49 (49.5%)	9 (17.6%)	17 (33.3%)	25 (49.1%)
Avoid caffeinated drinks	15 (15.1%)	39 (39.4%)	45 (45.5%)	10 (19.6%)	20 (39.2%)	21 (41.2%)
Smoking	9 (9.1%)	66 (66.7%)	24 (24.2%)	2 (3.9%)	37 (72.5%)	12 (23.6%)
Sleep 6–8 h daily	15 (15.2%)	21 (21.2%)	63 (63.6%)	5 (9.8%)	8 (15.7%)	38 (74.5%)
Sleep early and wake early	14 (14.1%)	12 (12.1%)	73 (73.8%)	8 (15.7%)	7 (13.7%)	36 (70.6%)
Engage in physical activity	16 (16.1%)	38 (38.4%)	45 (45.5%)	16 (31.4%)	23 (45.1%)	12 (23.5%)
Manage stress and emotions	32 (32.3%)	32 (32.3%)	35 (35.4%)	18 (35.3%)	17 (33.3%)	16 (31.4%)

**Table 3:** Percentage distribution of homeless adults’ knowledge about diabetes (n = 150).

Variables	Diabetic (n = 99)			Non-Diabetic (n = 51)		
	Incorrect	Correct Incomplete	Correct Complete	Incorrect	Correct Incomplete	Correct Complete
Definition of diabetes	82 (82.8%)	0 (0%)	17 (17.2%)	42 (82.4%)	0 (0%)	9 (17.6%)
Factors leading to diabetes	44 (44.4%)	55 (55.6%)	0 (0%)	30 (58.8%)	21 (41.2%)	0 (0%)
Symptoms of diabetes	50 (50.5%)	49 (49.5%)	0 (0%)	38 (74.5%)	13 (25.5%)	0 (0%)
Tests used to detect diabetes	57 (57.6%)	42 (42.4%)	0 (0%)	41 (80.4%)	10 (19.6%)	0 (0%)
Treatment of diabetes	42 (42.4%)	57 (57.6%)	0 (0%)	32 (62.7%)	19 (37.3%)	0 (0%)
Complications of diabetes	51 (51.5%)	48 (48.5%)	0 (0%)	40 (78.4%)	11 (21.6%)	0 (0%)
Prevention of diabetes	22 (22.2%)	77 (77.8%)	0 (0%)	21 (41.2%)	30 (58.8%)	0 (0%)

**Table 4:** Physiological measurements of homeless adults (n = 150).

Variables	Diabetic (n = 99)	Non-Diabetic (n = 51)	Chi-Square	df	*p*-Value
**BMI (kg/m^2^)**			21.987	6	0.001 **
<18.5	2 (2.1%)	5 (9.8%)			
18.5–24.9	20 (20.2%)	22 (43.1%)			
25–29.9	40 (40.4%)	13 (25.5%)			
≥30.0	37 (37.3%)	11 (21.6%)			
Mean ± SD	27.78 ± 4.06	24.93 ± 4.80			
**Waist circumference (cm)**			3.694	2	0.015 *
<85 in women and <90 in men	19 (19.2%)	17 (33.3%)			
≥85 in women and ≥90 in men	80 (80.8%)	34 (66.7%)			
Mean ± SD	99.3 ± 27.57	94.60 ± 24.5			
**TSF (mm)**			1.944	4	0.746
<10 in men and <15 in women	3 (3.1%)	2 (3.9%)			
10–15 in men and 15–25 in women	29 (29.3%)	20 (39.2%)			
>15 in men and >25 in women	67 (67.6%)	29 (56.9%)			
Mean ± SD	27.7 ± 19.03	21.76 ± 6.46			
**Blood pressure level (mm Hg)**			3.927	1	0.048 *
120/80–140/90	14 (14.1%)	14 (27.5%)			
>140/90	85 (85.9%)	37 (72.5%)			
Mean ± SD	131/82 ± 52/14	120/75 ± 11.2/8.6			

* Significant at <0.05; ** highly significant at <0.01.

**Table 5:** Comparison of model performance before and after feature selection.

	Before Feature Selection					After Feature Selection				
Model	Acc	Prec	Rec	F1	AUC	Acc	Prec	Rec	F1	AUC
Logistic Regression	0.63	0.71	0.67	0.69	—	0.70	0.82	0.70	0.76	—
SVM	0.53	0.62	0.73	0.67	—	0.57	0.64	0.80	0.71	—
Random Forest	0.67	0.72	0.75	0.73	—	0.70	0.76	0.80	0.78	—
Decision Tree	0.60	0.67	0.70	0.68	—	0.63	0.71	0.75	0.73	—
KNN	0.57	0.70	0.54	0.61	—	0.63	0.80	0.60	0.69	—
Gradient Boosting	0.60	0.68	0.65	0.67	—	0.63	0.74	0.70	0.72	—
**Hybrid Meta-Learning**	**0.82**	**0.89**	**0.80**	**0.84**	**0.87**	**0.95**	**1.00**	**0.94**	**0.97**	**0.98**

**Table 6:** Hyperparameter tuning and optimal configurations of machine learning algorithms.

Algorithm	Hyperparameters Tuned	Optimal Values
Logistic Regression	penalty (L1, L2, elasticnet); C (0.01, 0.1, 1, 10)	L2 penalty, C = 1.0
SVM	kernel (linear, RBF, polynomial); C (0.1, 1, 10); gamma (scale, auto)	RBF kernel, C = 10, gamma = scale
Random Forest	n_estimators (50, 100, 200); max_depth (3, 5, 7, 10); min_samples_split (2, 5, 10)	n_estimators = 100, max_depth = 5, min_samples_split = 5
Decision Tree	max_depth (3, 5, 7, 10, none); min_samples_split (2, 5, 10); criterion (Gini, entropy)	max_depth = 5, min_samples_split = 5, criterion = Gini
KNN	n_neighbors (3, 5, 7, 9, 11); weights (uniform, distance); metric (Euclidean, Manhattan)	n_neighbors = 7, weights = distance, metric = Euclidean
Gradient Boosting	n_estimators (50, 100, 200); learning_rate (0.01, 0.1, 0.2); max_depth (3, 5, 7)	n_estimators = 100, learning_rate = 0.1, max_depth = 3
XGBoost (Meta-Learner)	n_estimators (50, 100); learning_rate (0.01, 0.1); max_depth (3, 5)	n_estimators = 100, learning_rate = 0.1, max_depth = 3

**Table 7:** Confusion matrix components (TP, TN, FP, FN) for evaluated models.

Model	TP	TN	FP	FN
Logistic Regression	14	7	3	6
SVM	16	1	9	4
Random Forest	16	5	5	4
Decision Tree	15	4	6	5
KNN	12	7	3	8
Gradient Boosting	14	5	5	6
Hybrid Meta-Learning	15	13	0	2

Note: The test set contained 30 observations. TP = true positive, TN = true negative, FP = false positive, FN = false negative. The confusion matrix indicates that the hybrid model attained flawless precision (zero false positives) and substantial recall (merely 2 false negatives), accurately identifying 15 out of 17 diabetic cases and all 13 non-diabetic cases. The elimination of false positives is especially advantageous in screening applications, as unnecessary confirmatory testing can impose a burden on both patients and healthcare systems. The two overlooked diabetic patients (false negatives) exhibited atypical presentations characterized by a reduced BMI and blood pressure values, indicating possible constraints in identifying early-stage diabetes when conventional risk markers are not yet apparent.

## Data Availability

The data presented in this study are not publicly available due to ethical and privacy considerations involving homeless adults. Anonymized data may be made available from the corresponding author upon reasonable request and subject to approval by the relevant institutional and ethical committees.

## Abbreviations

The following abbreviations are used in this manuscript:

T2D	Type 2 Diabetes
BMI	Body Mass Index
TSF	Triceps Skin Fold Thickness
BSL	Blood Sugar Level
SBP	Systolic Blood Pressure
DBP	Diastolic Blood Pressure
LR	Logistic Regression
SVM	Support Vector Machine
RF	Random Forest
DT	Decision Tree
KNN	K-Nearest Neighbors
GB	Gradient Boosting
SMOTE	Synthetic Minority Over-Sampling Technique
SD	Standard Deviation
TP	True Positive
TN	True Negative
FP	False Positive
FN	False Negative
